# Characterization of an Ovine Bilateral Critical Sized Bone Defect Iliac Wing Model to Examine Treatment Modalities Based on Bone Tissue Engineering

**DOI:** 10.1155/2014/250958

**Published:** 2014-02-16

**Authors:** Jennifer L. Lansdowne, Declan Devine, Ursula Eberli, Pieter Emans, Tim J. M. Welting, Jim C. E. Odekerken, Damiano Schiuma, Martin Thalhauser, Ludovic Bouré, Stephan Zeiter

**Affiliations:** ^1^AO Research Institute Davos, Clavadelerstrasse 8, 7270 Davos Platz, Switzerland; ^2^Department of Orthopaedic Surgery, CAPHRI School for Public Health and Primary Care, Maastricht University Medical Centre, 6202 AZ Maastricht, The Netherlands

## Abstract

Critical sized bone defect (CSBD) animal models are used to evaluate and confirm efficacy and potency of new treatment modalities based on bone tissue engineering before the latter can be applied in clinical practice. In this study, a bilateral CSBD model in the iliac wings of sheep is described in detail. To demonstrate that this is a large animal CSBD model in sheep, bone healing within the defect left empty (negative control) or filled with autologous corticocancellous bone graft (clinical gold standard, positive control) was assessed using micro-CT, histology, histomorphometric, and fluorochrome analysis. After three months, new bone into the defect site was formed across the whole defect in the positive controls but limited to the edge of the defects in the negative controls. Bone volume in the positive controls was statistically higher than in the negative controls, with the latter having less than 10% new bone growth. There were no intraoperative or postoperative complications. The model described here represents a reliable and reproducible bilateral CSBD in sheep with low morbidity that can be used for *in vivo* evaluation of new treatment modalities based on bone tissue engineering.

## 1. Introduction

Before new treatment modalities based on bone tissue engineering can be used in clinical practice, their efficacy and potency require confirmation and evaluation in preclinical *in vivo* experiments, which often requires the use of a critical sized bone defect (CSBD) animal model [1–3]. A CSBD is defined as the smallest bone defect in a particular bone and species of animal, which will not heal spontaneously during the lifetime of that animal [1, 4]. More specifically, a CSBD has been described as a defect that has less than 10 percent bony regeneration during the lifetime of the animal [2] or duration of the experiment [5]. CSBDs should result in the formation of fibrous connective tissue rather than bone when left untreated (negative control), so that the osteogenic potential of the material being tested can be considered unequivocal [2]. Furthermore, CSBDs should heal when treated appropriately, that is, with the current gold standard (autologous bone graft, positive control). Any new treatment based on bone tissue engineering has to be evaluated against these two landmarks. Additionally, the ideal CSBD animal model creates little to no animal morbidity, has a low risk of complications, can provide more than one defect per animal in order to reduce the number of animals, can be imaged easily with advanced imaging techniques, and is reproducible. Last but not least, the defects should be of relevant size for neovascularization to occur, since vascularization remains one of the primary obstacles in the repair of bone defects [6, 7].

The os ilium can be used as a model for long bone defects because it forms by endochondral ossification [4]. Since it contains cortical and cancellous bone, it is comparable to the metaphyseal region of long bones. Moreover, it can be applied as a bilateral model and does not require any internal fixation to stabilize the defects, because it is not directly load bearing. The lack of internal fixation reduces the risk of postoperative complications and permits excellent imaging of the defect using computed tomography (CT) analysis by avoiding metallic artifacts. CSBDs in the os ilium (iliac wing, iliac crest) have already been described in the goat [4, 8]. Anderson et al. used adult female Dutch milk goats, to describe a bilateral iliac wing defect model using circular 17 mm defects with a three-month endpoint [4]. They report on histomorphometry that 13.5% of empty defects were filled with bone originating from the rim with loose connective tissue and fatty tissue filling the remainder of the defect, whereas defects filled with particulate autologous bone graft were almost completely bridged [4].

There is little information available in comparing the utility of goats versus sheep, and thus the choice of which small ruminant to use will depend on personal and institutional capabilities and on availability [9]. However, based on the number of publications, the sheep was more often used in orthopedic studies compared with the goat, but the CSBD in the os ilium has not been described in the sheep to date [10, 11]. Therefore, the objective of this study was to characterize in detail the bilateral 17 mm CSBD model in the iliac wings of sheep. To demonstrate that this is a large animal CSBD model in sheep which can be used for *in vivo* evaluation of new treatment modalities based on bone tissue engineering, bone healing within the defect left empty or filled with autologous bone graft was assessed using micro-CT, histology, histomorphometric, and fluorochrome analysis.

## 2. Materials and Methods

### 2.1. Animals and Treatment Randomization

Six adult female Swiss alpine white sheep were used in this study. Their age ranged between 2.5 and 3.5 years and their weight was 71.9 ± 2.2 kg (mean ± SD). The sheep were assessed to be healthy and free of diseases based on clinical examination and complete blood cell count prior to inclusion in the study. Two weeks before the surgery the animals were moved to the research facility for acclimatization. Group size was continuously decreased so that animals were housed in single stalls at the end of the acclimatization period. The sheep remained in individual stalls for two weeks after surgical intervention, after which they were group housed until euthanasia. All procedures were approved by the Graubünden Animal Commission and performed in an approved facility in accordance with the Swiss Animal Protection Law.

Each sheep received two CSBDs, one in each iliac wing. All sheep had a positive and a negative control defect, randomly assigned to either the right or left side for a total of 6 negative and 6 positive control defects. Negative control defects were left empty and positive control defects were filled with the morselized corticocancellous bone plug removed to create the contralateral negative control defect. All sheep were humanely euthanized after three months by intravenous injection of pentobarbital (0.5 mL/kg; Esconarkon, Ad. Us. Vet.).

### 2.2. Anesthesia and Analgesia Protocols

Standard operating protocols for anesthesia and perioperative analgesia were used. The procedure to create the iliac crest defects was performed with the sheep placed under general anesthesia. The animals were premedicated with diazepam (Valium) 0.3 mg/kg intravenous (IV) and ketamine (Ketasol-100) 2 mg/kg IV and general anesthesia was induced with propofol (Propofol 1% Fresenius) 2 mg/kg IV until the animal could be intubated with a cuffed endotracheal tube (9.5 or 10 I.D. mm, Rüschelit). General anesthesia was maintained with isoflurane at approximately 2% in oxygen with an oxygen flow rate of 500 mL/kg/min. Spinal intrathecal anesthesia was performed with the administration of 0.05 mg/kg xylazine (Rompun) at the lumbosacral space or between the second to last and last lumbar vertebrae and a local infiltration with lidocaine (Ultracain, 3 mL) was performed over the intended surgical site prior to incision. Postoperative carprofen (Rimadyl) 4 mg/kg subcutaneous (SC) once daily, for three days, plus buprenorphine (Temgesic) 0.1 mg/kg intramuscularly (IM), three times daily for one day, and a fentanyl patch (Fentanyl Patch Janssen, 2 mcg/kg/hr) for 72 hours (effective 12–24 hours after surgery) were all started at the completion of the surgical procedure before the sheep was recovered from anesthesia.

### 2.3. Surgical and *In Vivo* Procedures

**Figure 1:**
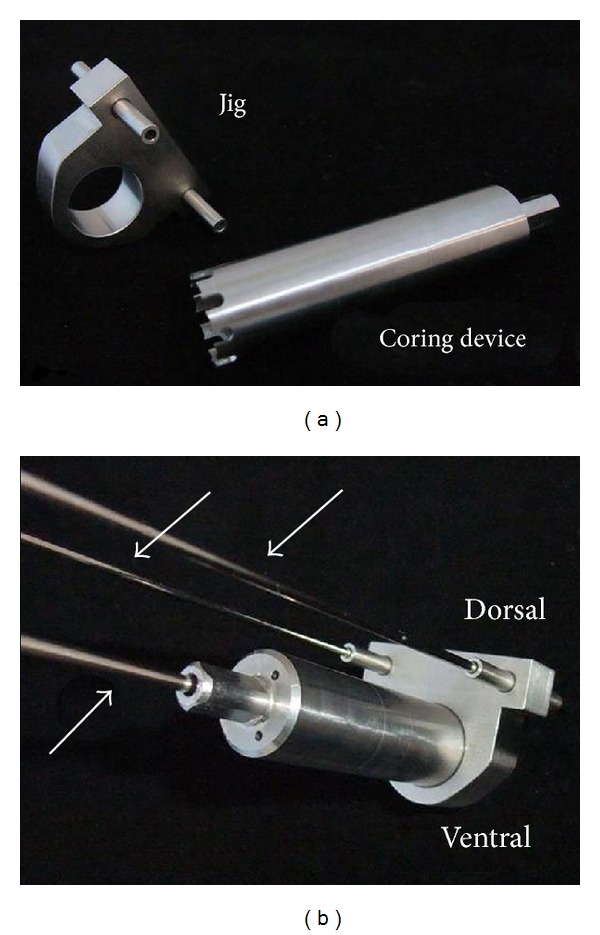
(a) Photograph of the custom-made jig and the 17 mm coring device used to create the defect. (b) Orientation of the jig with two 2.0 mm K-wires located dorsally (top white arrows) and one 2.5 mm K-wire located centrally through the coring device which is subsequently placed through the jig (bottom white arrow).

**Figure 2:**
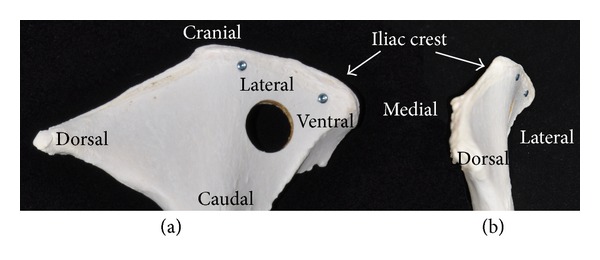
Photograph of a bone specimen from a Swiss alpine white sheep showing the anatomical orientation (a and b) and the cranial ventral locating screws along the iliac crest and central 17 mm defect (a).

**Figure 3:**
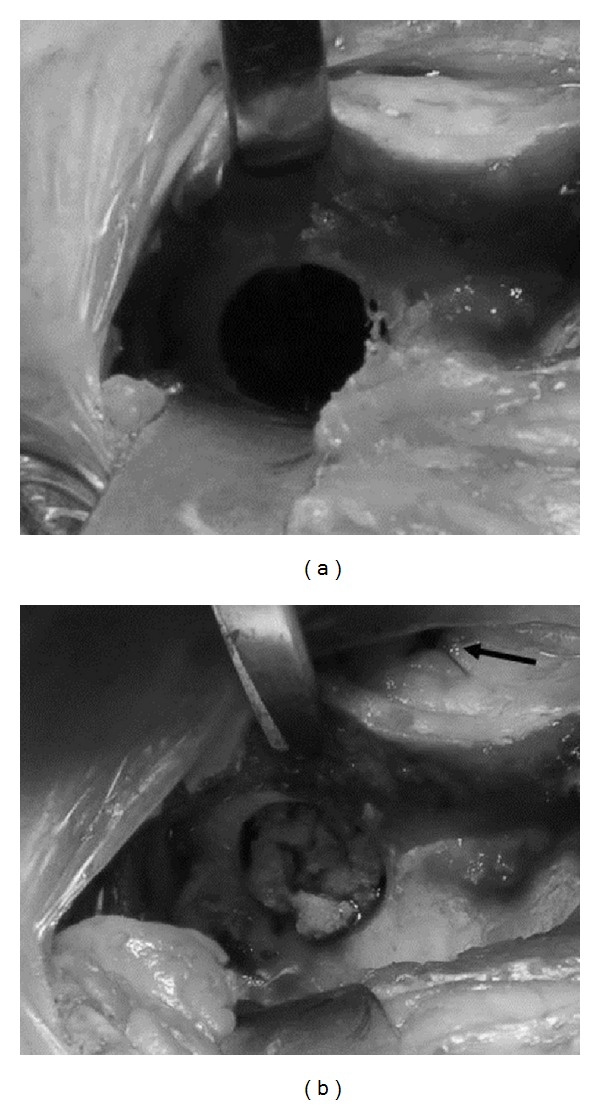
(a) Resulting empty defect. (b) Morselized corticocancellous bone graft stacked within the defect. Note the closure at the craniomedial aspect (black arrow).

Sheep were placed in sternal recumbency with their hind legs pulled cranially. The area from approximately lumbar vertebrae two to the tail head and on both sides to the level of the coxofemoral joints was clipped, prepped, and draped for sterile surgery. A curved incision was made ventrodorsally over the cranial aspect of one iliac crest through the skin and subcutaneous tissue and extended down to the periosteum. Langenbeck and Gelpi retractors were used to provide tissue retraction. The middle gluteal muscle was sharply transected parallel to the iliac crest and 1 cm from its attachment to the crest. The periosteum and middle gluteal muscle were elevated to expose the lateral surface of the iliac wing. Medially, the fascia was sharply transected 5 mm cranially and parallel to the iliac crest and the combined iliocostalis and longissimus lumborum muscles were elevated from the periosteum on the medial surface of the iliac wing using a periosteal elevator. A 2 cm wide malleable retractor was placed between the detached iliocostalis and longissimus lumborum muscles and the medial surface of the iliac wing to provide protection during defect creation. A custom-made surgical jig, a 17 mm diameter coring device, and three K-wires were used to create the defects. The surgical jig was positioned on the exposed lateral surface of the iliac wing using three K-wires. Two cranially located 2.0 mm K-wires were placed and one central 2.5 mm K-wire was placed (Figure 1). The jig was positioned so that the cranial and ventral sides of the created defect were surrounded by at least a 1 cm bony rim (Figure 2). The jig and coring device were then removed and a screw (2.4 mm self-tapping titanium; Synthes) was placed at each cranial location along the iliac crest after removal of the 2.0 mm K-wire to act as locating screws during *ex vivo* defect harvesting. The 17 mm coring device was rethreaded over the central 2.5 mm K-wire to help centralize the defect and the hole was drilled. During drilling there was constant cooling of the coring device with sterile physiologic 0.9% NaCl solution. Following defect creation the periosteum remaining on the medial aspect was removed using a combination of sharp transection through the defect and elevation from the medial surface. Additionally the area was irrigated. The medial tissues were closed by opposing the fascia of the iliocostalis and longissimus lumborum muscles using 0 poliglecaprone 25 (Monocryl, Ethicon) in a simple interrupted or cruciate suture pattern. For defects receiving autologous bone grafts the 17 mm bone core removed from the contralateral side was morselized with bone rongeurs, mixed with blood from the surgical site, and placed by stacking bone fragments into the defect (Figure 3(b)). The lateral periosteum was removed from the medial surface of the gluteal muscle using Metzenbaum scissors. Afterwards, the middle gluteal muscle and subcutaneous tissue were sutured over the iliac crest routinely in layers (poliglecaprone 25; 0 Monocryl, 2-0 Monocryl, Ethicon) followed by skin sutures or staples and an adhesive bandage (glue-on sterile antimicrobial drapes; Ioban 2). The same procedure was used to create an identical defect in the contralateral iliac wing. Bandages were removed 3–5 days postoperatively and sutures or staples were removed 14 days postoperatively.

The sheep were checked three times a day by an experienced animal caregiver for the first two postoperative weeks, followed by twice daily until the end of the experiment. Body weight was recorded monthly. All animals received fluorochrome labelling with calcein green (10 mg/kg, SC) at three weeks, xylenol orange (90 mg/kg, SC) at six weeks, and oxytetracycline (30 mL per animal, SC) at nine weeks after surgery to assess the amount of bone formed at different stages of healing. The animals were humanely euthanized by intravenous administration of pentobarbital (0.5 mL/kg; Esconarkon, Ad. Us. Vet.) after three months. Postmortem the soft tissues were immediately removed from the iliac crest and the defects were cored out using a 25 mm coring device utilizing a custom made jig fixed in position using the locating screw holes placed during the original surgery. The explanted samples were fixed in 70% ethanol for one week prior to micro-CT scanning.

### 2.4. *Ex Vivo* Analysis

#### 2.4.1. Micro-Computed Tomography (Micro-CT) Evaluation

Postmortem micro-CT measurements (*μ*CT40, SCANCO Medical AG) of the defects were performed to quantify the volume of material in the defect. The harvested samples were placed in a holding device filled with 70% ethanol and securely closed. Computed tomography measurements were performed at 70 kVp, 114 *μ*A, and isotropic resolution of 36 *μ*m. The volume of interest consisted of the entire thickness and diameter (17 mm) of the original defect. A threshold of 400 mgHA/cm^3^ was used to separate bone from bone marrow. Mean bone volume (mm^3^) and mean bone density (mg HA/cm^3^) for positive controls and negative controls were determined and representative three-dimensional reconstruction images of samples analyzed were created.

#### 2.4.2. Histological and Histomorphometric Analysis

For histological processing, samples were fixed in 70% ethanol for approximately six weeks. The samples were then dehydrated in an ascending alcohol series, cleared in an intermedium solvent (xylene) for 1–5 days, prior to infiltration with and embedding in methyl methacrylate (MMA). The samples were trimmed with a band saw to remove excess MMA so that they could be placed into a Leica 1600 saw (Leica AG). Serial cross sections approximately 200 *μ*m thick were prepared. Contact microradiographs of each section were taken using a cabinet X-ray system (model 43855A, Faxitron X-ray Corporation). These radiographs were used to select representative sections for further analysis. The selected sections were glued to Plexiglass using cyanoacrylate, ground, and polished to a thickness of 60–100 microns using an Exact Micro Grinding System.

**Figure 4:**
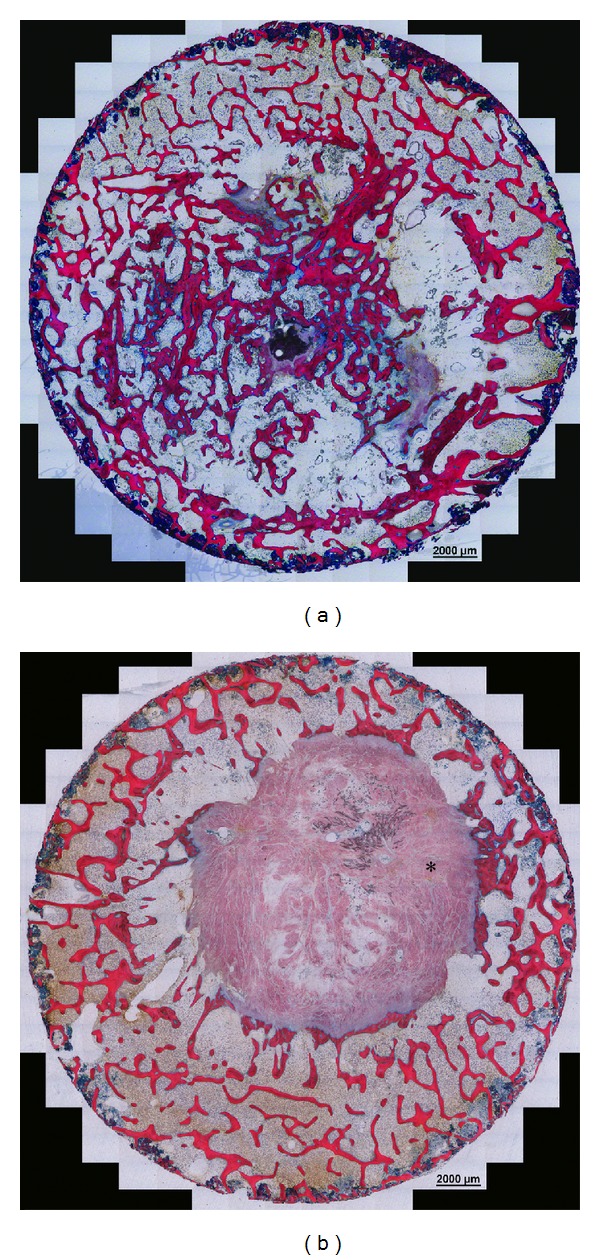
Photomicrograph of a positive control defect (a) and negative control defect (b) within the ROI (white circle). New bone growth into the positive control defect was widespread, while new bone growth into the empty defect occurred restricted to the edge of the defect. Dense connective tissue was the major tissue in the empty defect (∗). The sections were stained with Giemsa-eosin.

For histomorphometric analysis, sections stained with Giemsa-eosin (GE) were digitized using a Laser Scanning Microscope (Axiovert 200M) at an objective setting of 5X. These images were digitally combined to recreate the entire defect in photomicrograph form. In each photomicrograph, a region of interest (ROI) was defined which consisted of a 17 mm diameter circle drawn around the edge of the former defect (Figure 4). Relative bone formation within the ROI was assessed with the aid of histomorphometry software (KS400 version 3.0, Zeiss). Results were presented as the percent of bone per ROI.

For qualitative evaluation of bone formation over time, analysis with fluorochromes was performed with unstained sections.

#### 2.4.3. Statistical Analysis

Statistical evaluation was performed using SPSS for Windows (version 18). A paired *t*-test was performed to detect significant differences between treatment groups. For all statistical tests *P* ≤ 0.05 was considered to be significant.

## 3. Results

All preoperative complete blood count results and physical examinations were within normal ranges for healthy sheep. There were no intraoperative complications. The periosteum was difficult to remove from the medial aspect of the ilium, but it was accomplished in all cases using a combination of sharp dissection and elevation. The tissue appeared to naturally contact the defect surface on the medial aspect of the ilium, but a space remained laterally. Although no tissue was specifically apposed over the defect the graft was stable and in no case did it migrate or became dislodged. All sheep recovered uneventfully from anesthesia and surgery. They were able to fully weight bear on all limbs immediately after recovery. The surgical incisions healed uneventfully. No abnormalities were recorded during any daily health check. Mean animal weight just prior to euthanasia was 72 ± 2.7 kg.

**Figure 5:**
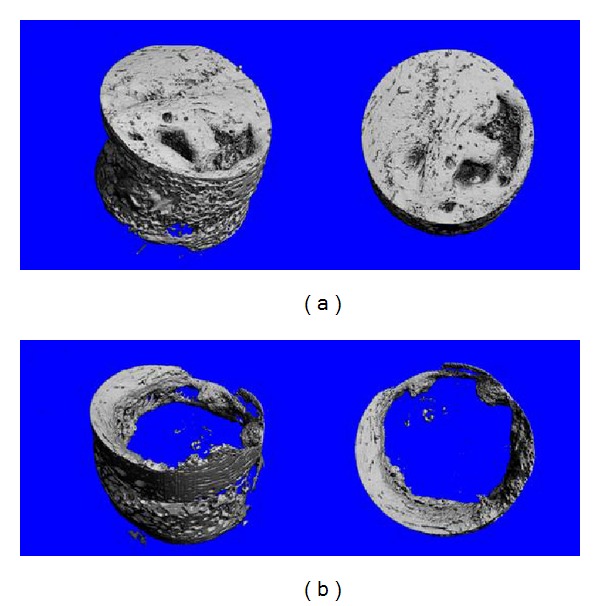
Representative micro-CT 3D reconstructions of positive control defects (treated with autologous bone) (a) and negative control defects (left empty) (b).

Representative three-dimensional reconstruction images of samples analyzed using micro-CT are shown in Figure 5. Mean bone volume and bone density are presented in Table 1. Bone volume in the positive control defects was statistically higher than in the negative control defects (*P* = 0.001). There was no difference in bone density of newly formed bone between positive and negative controls (*P* = 0.451).

GE stained sections were assessed for tissue in the defect and for signs of bone formation and remodeling. The major tissue in positive control samples was bone. In the center of the defect only some dense connective tissue was present (Figure 4(a)). The bone varied from immature bone which was undergoing remodeling as indicated by osteons and some osteoclasts, to mature trabecular bone. Active bone formation was ongoing as indicated by the presence of osteoid.

The major tissue in the negative control samples was dense connective tissue and bone formation was limited to the edge of the defects (Figure 4(b)). Bone remodeling was observed through the presence of some osteons and evidence of osteoclasts (Howship's lacunae).

Histomorphometric analysis revealed that the percentage of bone in the defect was 22.18 ± 8.48% (mean ± SD) for the positive control defect, which was statistically higher than in the empty defects (9.52 ± 2.67%; *P* = 0.009).

**Figure 6:**
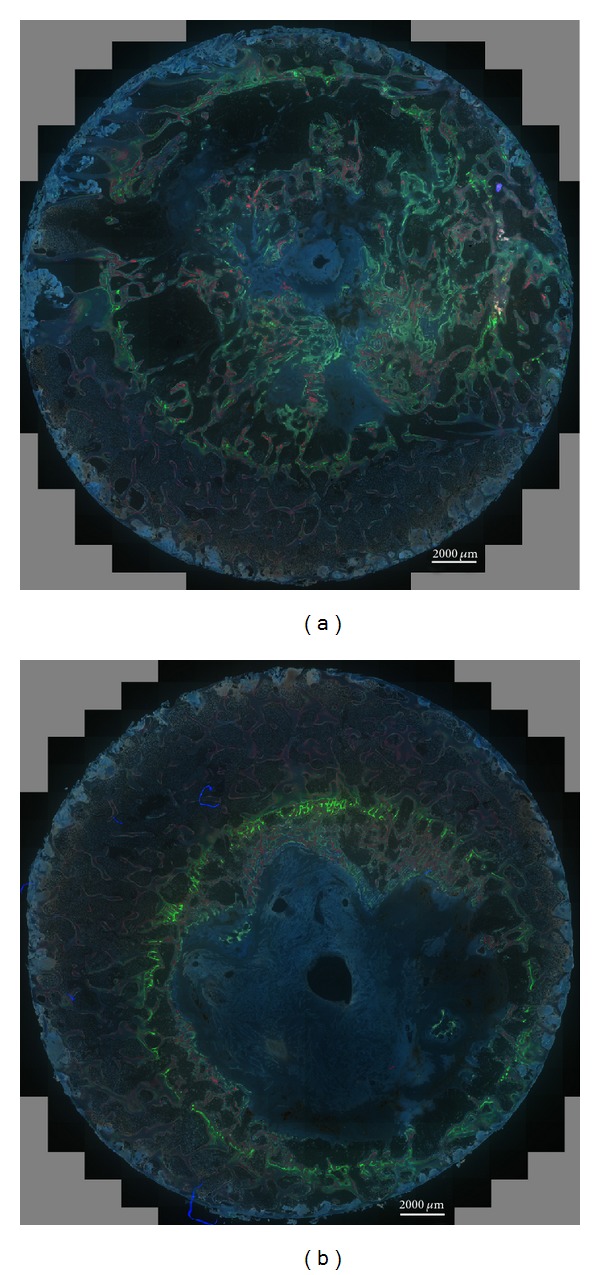
Fluorochrome images of positive control defect (a) showing the widespread new bone growing into the defect and negative control defect (b) showing that the fluorochromes are confined to the edges with minimal bone growth after 3 weeks. Fluorochromes were given at three weeks (calcein green), at six weeks (xylenol orange), and at nine weeks after surgery (oxytetracycline).

In the positive control defects the calcein green was mainly visible at the outer edge of the defect. This indicates that minor bone formation occurred during the first three *in vivo* weeks. Xylenol orange was also visible at the edge of the defects, but it was most prominent within the defect indicating good bone formation between three and six weeks. Oxytetracycline was prominent in the defect and was seen towards the center of the defect. There was also evidence that bone formation occurred after the oxytetracycline administration. In the negative control defects calcein green was visible mainly at the outer edge of the defect. Xylenol orange was also visible at the outer edge of the defects and to a limited extent within the defect but was not widespread. There was minimal bone formation after oxytetracycline administration. As bone formation into the defect was limited, the three fluorochrome labels were in close proximity to each other especially at the outer edge of the defect in negative control samples (Figure 6).

## 4. Discussion

CSBD models have been used to assess new treatment modalities based on bone tissue engineering intended to repair or regenerate bone. Many different models in different species have been described [12, 13]. The careful choice of model will mainly depend on the research question or intended clinical application, as well as on personal and institutional capabilities, experiences, and preferences. Nevertheless, there is an ethical, scientific, and economical imperative, that all models are well defined and standardized in order to reduce variation, and hence, reduce number of animals and resources needed and to maximize validity of the obtained results. This study is not meant to exclude other models and/or methods but rather intended to provide researchers characteristics, advantages, and limitations of this model, which might be useful to investigate tissue engineered constructs in this large animal model.

The model described fulfills the main criteria of a CSBD model as follow.

- When left untreated, the major tissue in the negative control samples was dense connective tissue and bone formation was limited to the border of the defect. For this reason the osteogenic potential of the material being tested can be considered unequivocal. At the 3-month time-point there was less than 10% new bone in the empty defects, meeting another proposed requirement of a CSBD [2, 5].
- The created defects heal when treated with the current gold standard (autologous bone graft). Results indicate that the amount of bone in the positive controls is greater than in the negative controls suggesting almost complete healing of the defect. The histological review of the sections indicates that the bone in the defect was undergoing remodeling as a result of new bone formation, eliminating the possibility that the higher bone volume assessed in the positive controls was simply the actual bone graft placed at surgery.

Therefore, using this model, any new treatment based on bone tissue engineering can be evaluated against these two landmarks to assess its efficacy and potency. Moreover, the model has several advantages over existing large animal models.

- There is the ability to create two CSBDs within the same animal. This could reduce the number of animals required in a study, allow the comparison of two treatments within the same animal, or allow one defect to act as an internal control. In CSBD of long bones, bilateral models are generally considered contraindicated due to humane reasons and also possible effects on data integrity [12].
- All sheep tolerated the procedure very well. No intraoperative or postoperative complications occurred. With adequate analgesia, as provided in this study, sheep should be able to ambulate normally within 24 hours of the procedure.
- The surgical procedure is relatively easy when attention is given to important landmarks such as the cranial and ventral border of the iliac wing. From a surgical point of view these anatomical locations correspond to dorsal and lateral, respectively. Appropriate retraction facilitates exposure and limits soft tissue dissection. Using a surgical jig and a coring device the defect creation is reproducible and of consistent size. Products made to precisely fit the 17 mm defect have good bone contact and stability within the defect brackets (Bergmann et al., unpublished work). The area exposed using the technique described in this study could potentially hold a larger defect up to 25 mm as performed in cadaveric specimens, but this would need to be evaluated *in vivo*.
- It is an attractive model as results can be provided in a relatively short period of time (three months). Nevertheless, a longer observation period such as 6 months would be needed to determine the full extent of healing in the empty defect.
- There is no concern about leakage of materials from one site to the other as in calvarial defects, which can have a relatively close proximity to each other and a fluid continuum along the dura or subcutaneous tissues [14].
- This model requires no fixation over the defect, which reduces the risk of postoperative complications often seen in studies of CSBD in long bones of large animals. Further, it prevents artefacts, if advanced imaging such as CT scanning is intended.

Nevertheless, the following points need to be considered.

- The mechanical environment has been shown to affect tissue repair [15] and mesenchymal stem cell differentiation [16, 17]. Most published CSBD models to assess bone regeneration by endochondral ossification are stabilized segmental defects of long bones, which are weight bearing. Defect site and its stabilization will influence the local mechanical environment. The CSBD in the os ilium is not weight bearing and not stabilized. This has to be carefully considered, while the impact will depend on the intended clinical use of the tissue engineered construct.
- The os ilium contains cortical and cancellous bone and, therefore, it is comparable to the metaphyseal region of long bones. This difference to diaphyseal segmental long bone defects consisting of mainly cortical bone has to be taken into account. Compared with drill hole defects (e.g., in the distal femur) healing of larger sized bone defects can be assessed.
- In our experience taking meaningful *in vivo* radiographs of this area is difficult due to the orientation of the pelvis in the sheep.
- The treatment material must be self-retaining within the vertically oriented defect, which is round shaped. Stacking pieces of corticocancellous bone graft (as in the positive control in this study) worked well, but this model would not work for semiliquid or soft materials. Since the medial aspect of the defect has close tissue coverage, it is possible that a mesh or other protective barrier could be applied to the lateral aspect of the ilium to maintain soft products. However, this should be evaluated *in vivo*.

## 5. Conclusions

The model described here represents a low morbidity, reproducible bilateral critical sized bone defect in a large animal and can be used for *in vivo* evaluation of new treatment modalities based on bone tissue engineering which can be self-retaining in a vertically oriented round defect.

## Figures and Tables

**Table 1:** Comparison of bone volume and bone density between the two groups as determined by micro-CT analysis.

Group	Mean bone volume (mm^3^) (Mean ± SD)	Mean bone density (mg HA/cm^3^) (Mean ± SD)
Autograft—positive control	520.1 ± 123.8*	587.3 ± 17.3
Empty—negative control	224.3 ± 84.1*	576.5 ± 18.0

*Indicates significant differences.

## Abbreviations

CSBD: Criticalsized bone defect
IV: Intravenous
SC: Subcutaneous
IM: Intramuscular
CT: Computed tomography
MMA: Methyl methacrylate
GE: Giemsa-eosin
ROI: Region of interest
SD: Standard deviation.
